# Frequency-Multiplication Transponder Radar for Geological Hazard Deformation Monitoring

**DOI:** 10.3390/s26217054

**Published:** 2026-11-08

**Authors:** Ning An, Tao Wang, Jianjun Rao, Guoxuan Ding

**Affiliations:** 1 Gansu Institute of Engineering Geology, Gansu Provincial Bureau of Geology and Mineral Exploration & Development, Lanzhou 730030, China; dgx5603@163.com; 2 Center of Communication and Tracking Telemetering & Command, Chongqing University, Chongqing 400044, China; jianjunrao@163.com

**Keywords:** deformation monitoring, phase interferometry, frequency-multiplication transponder, pulse power amplifier

## Abstract

**Highlights:**

**What are the main findings?**

- A frequency-multiplication transponder radar was proposed to suppress transmitter leakage and amplify long-range echoes for micro-deformation monitoring.
- OOK frequency division enabled simultaneous multi-target displacement demodulation with 2.5 mm spatial resolution, <0.8 mm maximum error, and 0.14 mm RMSE.

**What are the implications of the main findings?**

- The proposed radar provides a practical, sub-millimeter, long-range monitoring solution for slopes, bridges, and large civil structures.
- This system addresses the key limitations of conventional phase-interferometric radar regarding measurement range, transmitter leakage, and multi-target identification.

**Abstract:**

Remote micro-deformation monitoring of large structures and geological hazards presents significant challenges for conventional phase-interferometric radar due to limitations in range, transmitter leakage, and multi-target identification. This study proposed a deformation monitoring radar utilizing a frequency-multiplication transponder. Frequency isolation effectively suppresses transmitter leakage, while active frequency-multiplication amplification enhances echo signals over long distances. Additionally, on–off keying (OOK) frequency division allows for the separation of multiple targets. A transponder interferometric model established a relationship between target displacement and echo phase. A prototype-measured link budget established the theoretical operational range limit. Each measurement point required a dedicated active transponder. Consequently, the system conducted sparse point-wise radial displacement monitoring rather than full-field imaging. Field experiments were performed to evaluate displacement measurement at 129 m, 345 m, and 570 m. A dual-target experiment was also conducted at 129 m. All test configurations successfully resolved 2.5 mm micro-displacements. The measurement error was approximately 0.1 mm under unobstructed conditions. The maximum absolute error remained below 0.8 mm in complex environments. Dual-target demodulation yielded a root-mean-square error of 0.14 mm. The maximum experimentally verified distance is 570 m. The 1 km design range represents a theoretical link-budget target. This target is constrained by the transponder saturation threshold and receiver sensitivity. Field validation at 1 km remains unachieved.

## 1. Introduction

Complex geological conditions across China frequently trigger severe geohazards, including landslides and rockfalls. These disasters directly threaten transportation networks, hydraulic facilities, and urban settlements. Accurate early warning requires an integrated “space–air–ground–interior” collaborative monitoring framework [1,2]. High-precision monitoring of micro-deformations in rock and soil masses constitutes a pivotal component. This type of monitoring requires remote, non-contact, sub-millimeter-level, and simultaneous multi-target observations. Nevertheless, the instability of rock and soil masses does not occur as a singular, instantaneous event; instead, it represents a gradual evolutionary process encompassing strain localization, segmented accumulation of damage, and ultimately through-slip prior to failure. Depending on the conditions of structural planes, various instability modes may emerge [3,4]. Consequently, high-precision displacement monitoring can elucidate the correlation between deformation behavior and internal damage evolution in rock and soil masses, capture localized precursor signs preceding instability, and thereby furnish a foundation for disaster assessment, hazard delineation, and preventive strategies.

Multiple technical systems exist for displacement monitoring [5]. However, each approach presents distinct limitations. Fiber-optic sensing offers high measurement accuracy. Nevertheless, this technique requires complex installation and intensive maintenance [6,7,8,9]. GNSS enables long-range monitoring. However, its precision and sampling rates remain insufficient for early micro-deformation identification [10,11,12,13]. Close-range photogrammetry delivers intuitive, non-contact spatial data [14,15]. Nonetheless, severe weather conditions significantly degrade its measurement accuracy. Three-dimensional laser scanning offers efficient and comprehensive data acquisition [16,17,18]. However, ambient factors such as illumination and temperature variations affect the results. In addition, the required instrumentation entails substantial capital costs.

Phase interferometry serves as a prominent technique for non-contact, all-weather, and high-precision deformation measurements [19,20,21]. However, conventional interferometric radar suffers from severe long-range echo attenuation. Transmitter leakage further degrades receiver sensitivity. Multi-target identification also presents significant operational challenges. These limitations impede comprehensive geological hazard monitoring. This study developed a deformation monitoring radar based on frequency-multiplication transponders to resolve these deficiencies. The proposed design integrates frequency isolation, active frequency-multiplication amplification, and OOK pulse modulation. The hardware design, measurement model, and derived relationships were verified by simulation and field experiments. Experimental results demonstrated sub-millimeter accuracy and simultaneous multi-target monitoring across distances from 129 m to 570 m. Link-budget evaluations indicated a component-limited operating range of approximately 1 km. This projected distance serves as a design target rather than an empirical measurement. Each target position required a dedicated transponder. Consequently, the system operated as a sparse point-type radial displacement monitor rather than a full-field imaging platform.

## 2. Materials and Methods

### 2.1. Radar System Configuration

**Figure 1:**
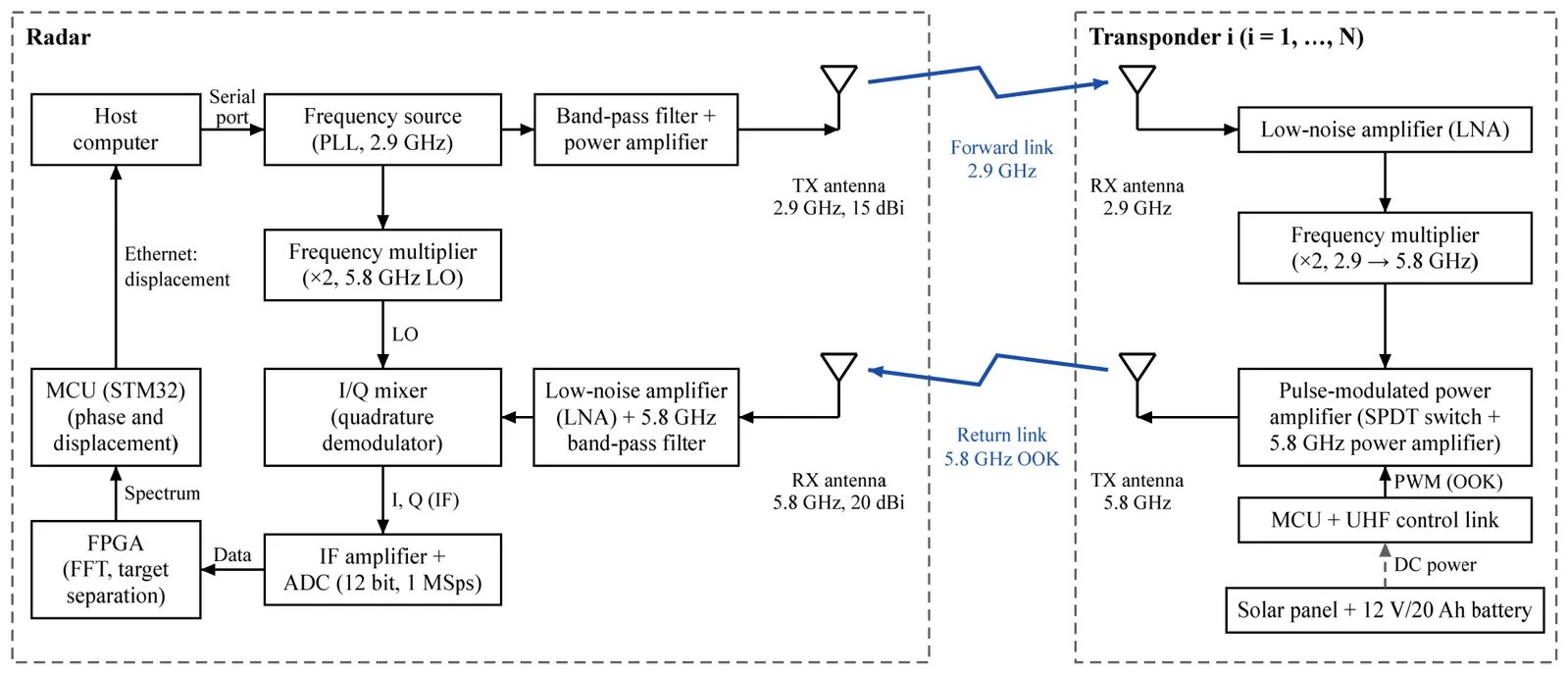
Block diagram of the radar system. Arrows denote the direction of signal propagation. All radio links operate in a unidirectional mode. The forward link connects the 2.9 GHz transmitting (TX) antenna of the radar to the receiving (RX) antenna of the transponder. The return link connects the 5.8 GHz TX antenna of the transponder to the RX antenna of the radar. PLL: phase-locked loop; LO: local oscillator; LNA: low-noise amplifier; IF: intermediate frequency; ADC: analog-to-digital converter; FFT: fast Fourier transform; FPGA: field-programmable gate array; MCU: microcontroller unit; SPDT: single-pole double-throw; PWM: pulse-width modulation; OOK: on–off keying; UHF: ultra-high frequency.

The proposed deformation monitoring radar consists of a radar transceiver and frequency-multiplication transponders. Figure 1 illustrates the overall hardware architecture. The system employs dedicated transmitting and receiving antennas. The transmitting antenna belongs to the transmit chain and radiates a 2.9 GHz carrier with a gain of 15 dBi. The receiving antenna operates in the receive chain and captures the 5.8 GHz echo with a gain of 20 dBi. These antennas operate in distinct frequency bands and are strictly non-interchangeable. All electromagnetic wave paths are strictly unidirectional. The radar transceiver includes an RF output module, a filtering and amplification module, a mixing module, a data acquisition and processing module, and an uploading module. Signal processing is performed jointly by a microcontroller unit (MCU) and a field-programmable gate array (FPGA).

The RF output module generates two 2.9 GHz signals from a single frequency synthesizer. One signal undergoes bandpass filtering and amplification. The transmitting antenna radiates this signal towards the transponder to establish the forward link. The second signal enters a frequency multiplication circuit and is converted to 5.8 GHz. Subsequent filtering and amplification condition this signal as the frequency-multiplied local oscillator (LO) reference for the mixing module. The transponder receives the 2.9 GHz carrier signal. A low-noise amplifier (LNA) restores the signal drive level. A frequency doubler then upconverts the signal to 5.8 GHz. A microwave switch alternates between the 5.8 GHz power amplifier and a matched load at the modulation frequency. The OOK-modulated 5.8 GHz echo is transmitted to the radar via the return link. The radar receiving antenna captures this return signal. The forward link operates at 2.9 GHz, and the return link operates at 5.8 GHz. The radar receiver is tuned to 5.8 GHz. The direct 2.9 GHz antenna leakage falls outside the receiver passband and is rejected by the front-end filters. In addition, the radar derives its multiplied local oscillator from the primary carrier source. This shared reference enables coherent measurement of the echo phase.

The radar receiver filters and amplifies the received 5.8 GHz echo signal. A mixer combines this signal with a 5.8 GHz frequency-multiplied LO signal. This mixing stage yields an intermediate-frequency (IF) signal containing the modulation data. A dual-channel data acquisition board amplifies and digitizes the IF signal. The FPGA performs an FFT on the in-phase (I) and quadrature (Q) signals. The STM32 microcontroller extracts the phase information and calculates the target displacement. The microcontroller transmits the processed data to the host computer via Ethernet.

### 2.2. Frequency-Multiplication Transponder Measurement Model

The frequency-multiplication-transponder-based deformation monitoring radar consists of a radar end and a frequency-multiplication transponder installed on the structure. One output path of the radar end provides an RF carrier signal at the frequency $f_{0}$, while the other path passes through a frequency multiplier and becomes the frequency-multiplied local oscillator signal $2f_{0}$. The transponder receives the RF signal $f_{0}$ and frequency-multiplies it into an RF carrier signal $2f_{0}$, which is re-transmitted back to the radar receiver. At the receiving end, the modulated signal is quadrature down-converted with the frequency-multiplied local oscillator signal, and the displacement of the corresponding monitoring point is calculated according to the principle of phase interferometry.

The measurement model relies on three core assumptions. First, the carrier signal and the frequency-multiplied local oscillator remain strictly coherent. Both signals originate from a single HMC830 synthesizer referenced to a common 50 MHz crystal oscillator. This shared source yields correlated phase fluctuations and enables common-mode noise cancellation during down-conversion. In contrast, two free-running synthesizers would introduce phase drift directly into the interferometric phase and dominate the total error budget. Second, frequency doubling employs a passive HMC189A doubler. Its phase-noise contribution affects both the carrier and the local oscillator. Consequently, this shared noise largely cancels out. The synthesizer exhibits a phase noise below −100 dBc/Hz at a 10 kHz offset. This value corresponds to a displacement-equivalent noise of approximately 0.03 mm over the 500 kHz intermediate-frequency bandwidth. This noise level remains well below the reported measurement accuracy. Third, each transponder generates its modulation waveform using an independent local clock in multi-target mode. Target separation is achieved through an FFT with a bin spacing of 976.6 Hz. The clock drift over the observation window must remain well below one frequency bin. This condition requires a frequency offset below approximately 0.1 Hz for observation intervals between 60 s and 120 s. The crystal-controlled oscillators satisfy this requirement. Consequently, direct clock synchronization between the radar and transponders is not required.

The complex exponential signal transmitted by the radar is defined as:

$$s_{t}(t)=A_{t}e^{j(\omega_{0}t+\phi_{0})}$$

where $A_{t}$ is the signal amplitude, $\omega_{0}$ is the signal angular frequency $2\pi f_{0}$, and $\phi_{0}$ is the initial phase.

The signal propagates over a distance R to the transponder. The received signal contains additive white Gaussian noise:

$$s_{r}(t)=A_{r}e^{j\left(\omega_{0}\left(t-\frac{R}{c}\right)+\phi_{0}\right)}$$

where $A_{r}$ is the amplitude of the received signal and c is the speed of light in vacuum. Frequency multiplication increases the carrier frequency to $2f_{0}$. The corresponding equivalent wavelength becomes $\lambda_{0}/2$. Consequently, the phase change is doubled. The re-transmitted signal is expressed as:

$$s_{\mathrm{tr}}(t)=A_{\mathrm{tr}}m(t)e^{j\left(2\omega_{0}\left(t-\frac{R}{c}\right)+\phi_{0}\right)}$$

where $A_{tr}$ is the re-transmitted signal amplitude and m(t) is the OOK modulation signal.

The re-transmitted signal propagates along the two-way path back to the radar system. The received echo contains additive noise:

$$s_{\mathrm{rec}}(t)=A_{\mathrm{rec}}m(t)e^{j\left(2\omega_{0}\left(t-\frac{2R}{c}\right)+\phi_{0}\right)}+n_{\mathrm{rec}}(t)$$

where $A_{\mathrm{rec}}$ is the amplitude of the received signal and $n_{\mathrm{rec}}(t)$ is the total noise at the radar receiver. The noisy phase observation is expressed as follows:

$$\hat{\phi }=\frac{8\pi f_{0}R}{c}+\phi_{\mathrm{rec}}$$

where $\phi_{\mathrm{rec}}$ is the noise phase. The ΔR represents the target displacement, and Δϕ corresponds to the resulting phase variation. Let $\lambda_{0}=c/f_{0}$, the target displacement is derived as follows:

$$\Delta R=\frac{\lambda_{0}}{8\pi }\Delta \phi$$

The phase observation error $\sigma_{\phi }$ directly determines the displacement measurement error $\sigma_{\Delta R}$. This relationship is expressed as follows:

$$\sigma_{\Delta R}=\frac{\lambda_{0}}{8\pi }\sigma_{\phi }$$

The proposed system utilizes coherent reception. The recovered carrier exhibits phase jitter. Coherent communication theory defines the root-mean-square value of the phase jitter [22] as follows:

$$\sigma_{\phi }=\sqrt{\frac{1}{\mathrm{SNR}}}$$

where SNR is the signal-to-noise ratio of the received signal. The root-mean-square error of the displacement is defined as follows:

$$\sigma_{\Delta R}=\left(\frac{\lambda }{8\pi }\right)\sigma_{\phi }=\frac{\lambda }{8\pi }\sqrt{\frac{1}{\mathrm{SNR}}}$$

Arctangent demodulation restricts phase values to the interval [−π,π]. Phase variations beyond this boundary cause phase wrapping and result in erroneous displacement calculations. A single phase-wrapping step between consecutive solutions is approximately 12.9 mm. This value significantly exceeds normal slope displacement within a 0.1 s sampling interval. The host system evaluates each displacement increment against a 12.9 mm threshold. It also verifies normal operational ranges for both echo amplitude and SNR at the target modulation line. Failed measurements are classified as ambiguous and excluded from the cumulative displacement series. The system then alerts the operator to re-establish the reference. The unambiguous displacement is expressed as follows:

$$\Delta R_{\max}=\frac{\lambda_{0}}{4}$$

### 2.3. Multi-Target Displacement Measurement Model

The proposed method builds upon the conventional frequency-multiplication measurement model. Multiple OOK modulation signals with different frequencies are used to modulate the target signals of individual transponders. The radar receiver captures the superimposed multi-target echo signal. This signal undergoes frequency multiplication, OOK modulation, and power amplification. The resulting received signal is expressed as follows:

$$s_{\mathrm{rec}}(t)=\sum_{i=1}^{N}K_{i}\cdot p_{i}(t)\cdot e^{j\left(4\omega t+\phi_{i}+\varphi_{\Delta R,i}\right)}$$

where $p_{i}(t)$ is the OOK modulation signal of the i-th transponder; $K_{i}$ is the amplitude attenuation coefficient; $\phi_{i}$ is the fixed phase; $\varphi_{\Delta R,i}$ is the phase change caused by displacement; N is the number of targets, $\omega_{0}$ is the signal angular frequency $2\pi f_{0}$, and $\phi_{i}$ is the initial phase.

Quadrature down-conversion and low-pass filtering yield the baseband signal:

$$s_{\mathrm{bb}}(t)=\sum_{i=1}^{N}K_{i^{\prime }}\cdot p_{i}(t)\cdot e^{j\left(\phi_{i}+\varphi_{\Delta R,i}\right)}$$

The discrete baseband signal $s_{\mathrm{bb}}(n)$ is transformed by an $N_{\mathrm{FFT}}$-point FFT:

$$S[k]=\sum_{n=0}^{N_{\mathrm{FFT}}-1}s_{\mathrm{bb}}[n]e^{-j\frac{2\pi }{N_{\mathrm{FFT}}}kn}$$

The targets possess distinct modulation frequencies. Consequently, the signal energy concentrates at the corresponding frequency bin $k_{i}$ in the frequency domain. The complex spectral value of the i-th target is extracted from this bin:

S[ki]=ReS[ki]+j⋅ImS[ki]

The phase observation corresponding to this frequency bin is:

ϕ^i=arctanImS[ki]ReS[ki]

The displacement relationship in Section 2.1 yields the radial micro-displacement of the i-th target:

$$\Delta R_{i}=\frac{\lambda }{8\pi }\Delta {\hat{\phi }}_{i}$$

### 2.4. Monitoring Distance Analysis

The effective measurement range is a critical parameter in deformation monitoring applications [23]. The following derivation provides a detailed theoretical analysis of the radar monitoring distance.

Parameters $\Delta d^{\prime }$, $A_{1}$, and $A_{r}$ denote the displacement error threshold, the leakage signal amplitude, and the received signal amplitude, respectively. The corresponding maximum phase error $\Delta \varphi^{\prime }$ is expressed as follows:

$$\Delta \varphi^{\prime }=\frac{4\pi f\Delta d^{\prime }}{c}\leq \arcsin\left(\frac{A_{1}}{A_{r}}\right)$$

The leakage signal amplitude $A_{1}$ can be regarded as a constant under fixed transmit/receive antenna gains and relative radar positions. Throughout Equation (17) and the following expressions, the incremental one-way range change is written as Δd′, which is identical to the displacement ΔR defined in Equation (6); the corresponding phase increment Δφ′ is identical to Δφ, and the one-way range d corresponds to R in Section 2.2.

It is assumed that the transmit signal $S_{T}(t)$ has an input power of $P_{t}$. The transmitting and receiving antenna gains are denoted as $G_{t}$. The radar cross-section of the monitoring target is σ. The antenna receiving area is $S_{r}$. The standard radar equation defines the power of the echo signal $S_{r}(t)$ as follows:

$$P_{r}=P_{t}\cdot \frac{G_{t}\sigma S_{r}}{{\left(4\pi \right)}^{2}d^{4}}=P_{t}\cdot \frac{G_{t}^{2}\sigma \lambda^{2}}{{\left(4\pi \right)}^{3}d^{4}}$$

Rearrangement yields the maximum effective operating range $d_{\max}$ of the radar:

$$d_{\max}=\sqrt[{4}]{\frac{G_{t}\sigma S_{r}A_{t}^{2}}{{\left(4\pi \right)}^{2}A_{r}^{2}}}=\sqrt[{4}]{\frac{G_{t}\sigma \lambda^{2}A_{t}^{2}\sin^{2}\left(4\pi f\cdot \Delta d^{\prime }/c\right)}{{\left(4\pi \right)}^{3}A_{1}^{2}}}$$

Specifically, a ranging accuracy of $\Delta d^{\prime }$ corresponds to a maximum radar operating range of $d_{\max}$. According to Equation (19), increasing $d_{\max}$ at a fixed operating frequency requires either an increase in $G_{t}\sigma S_{r}A_{t}^{2}$ or a reduction in $A_{1}$. Device parameters limit $G_{t}$, $S_{r}$, and σ in the radar system. These metrics cannot be increased significantly under current hardware constraints. Consequently, increasing signal power and suppressing leakage serve as the primary approaches to extend the monitoring distance. Echo enhancement can adopt communication relay forwarding techniques. This enhancement is realized by integrating a power amplification module into an active backscatter transponder [24]. Furthermore, frequency isolation is an effective solution to reduce leakage [25]. Optimized antenna design and filtering achieve a coupling isolation exceeding 50 dB.

### 2.5. System Prototype and Experimental Setup

#### 2.5.1. System Prototype

**Figure 2:**
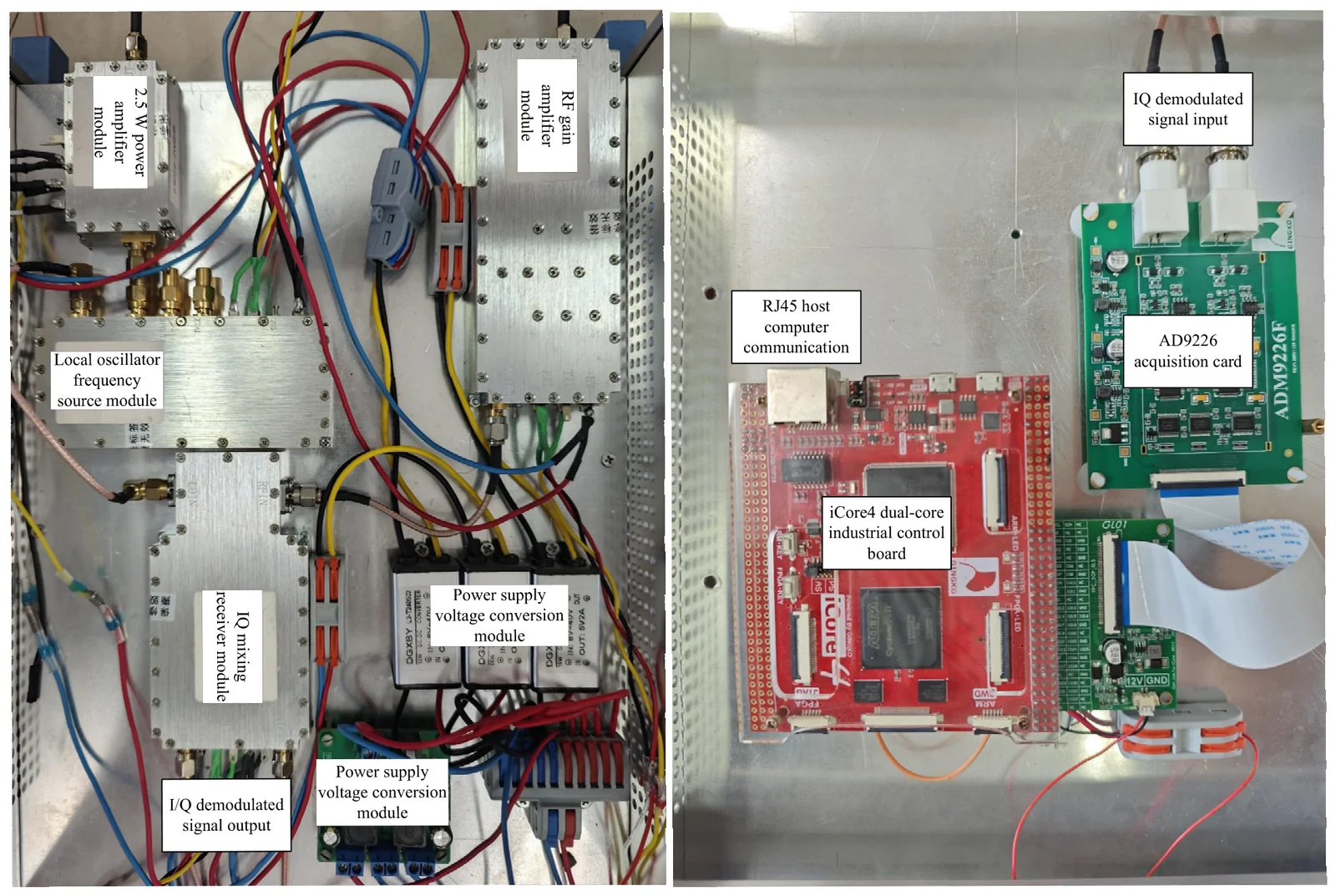
Internal architecture of the radar system. The left photo shows the radio-frequency subsystem. This subsystem comprises a 2.5 W power amplifier module, an RF gain amplifier module, an HMC830 local oscillator phase-locked loop synthesizer, an ADL5380 in-phase/quadrature (I/Q) demodulator, and voltage conversion modules. The right photo illustrates the data acquisition and processing subsystem. This subsystem contains an AD9226 ADC card and an iCore4 dual-core industrial control board, which integrates an FPGA and an STM32 microcontroller and communicates with the host computer through an RJ45 Ethernet interface.

**Figure 3:**
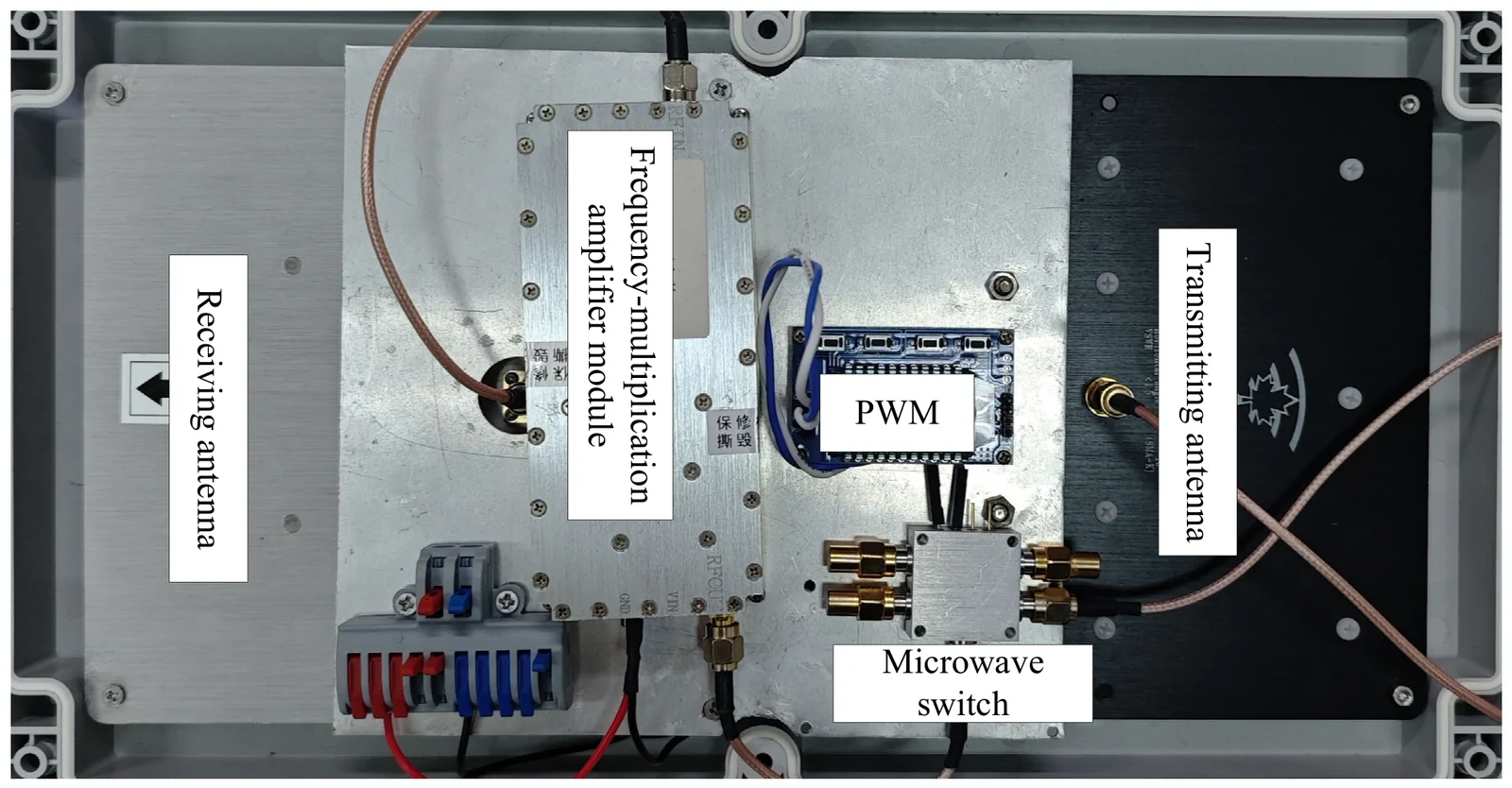
Internal architecture of the transponder. The frequency-multiplication amplifier module comprises a TQPM9037 low-noise amplifier (LNA) and an HMC189A frequency doubler. An HMC8038 single-pole double-throw (SPDT) microwave switch combines with an SE5004L 5.8 GHz power amplifier to constitute the pulse-modulated power amplifier. The pulse-width modulation (PWM) board supplies the on–off keying (OOK) modulation signal to the switch.

A hardware prototype was constructed using commercial off-the-shelf RF components for displacement monitoring verification. Figure 2 and Figure 3 illustrate the internal architectures of the radar and transponder units, respectively. The specific components of each module are detailed below for experimental reproducibility.

The radar frequency source utilizes an HMC830 fractional-N phase-locked loop synthesizer referenced to a 50 MHz crystal. This synthesizer generates the 2.9 GHz carrier signal. A host system controls the device via a CH340 serial interface. The carrier passes through a 2.9-GHz band-pass filter with a 1-MHz bandwidth. An RF power amplifier with an approximate gain of 30 dB then amplifies the signal. Consequently, the transmitting antenna radiates an output power of 33 dBm. In addition, the same source drives an HMC189A passive frequency doubler. The doubled output undergoes filtering and amplification to produce the 5.8-GHz frequency-multiplied local oscillator signal. This shared architecture ensures strict phase coherence between the carrier and the local oscillator. The received echo is amplified by a low-noise amplifier stage with an approximate gain of 55 dB. This stage is cascaded with a 5.8-GHz band-pass filter. A PE43703 seven-bit digital step attenuator regulates the signal power over a range of 0 to 30 dB. TCM1-63AX+ baluns convert the RF and local oscillator signals into differential inputs for an ADL5380 quadrature demodulator. An AD8130 difference amplifier converts the differential I and Q outputs to single-ended signals. An OPA365 intermediate-frequency (IF) stage with a 500-kHz bandwidth provides subsequent amplification. An AD9226 12-bit ADC samples both channels at rates up to 1 MSps. An iCore4 board integrates an FPGA and an STM32 microcontroller for system control. A LAN8720 Ethernet interface uploads the digital data at 100 Mbit/s. The measured receiver sensitivity is −80 dBm.

The transponder consists of a receiving antenna, a frequency-multiplication module, a pulse-modulated power amplifier, a control unit, and a power supply. A TQPM9037 low-noise amplifier provides a 16.5 dB gain and a 0.66 dB noise figure. It amplifies the received 2.9 GHz signal to the required input level for frequency doubling. An HMC189A passive doubler converts the signal to 5.8 GHz. This doubler exhibits approximately 15 dB conversion loss and over 30 dB fundamental and harmonic suppression. It introduces negligible residual phase noise. The pulse-modulated power amplifier integrates an HMC8038 single-pole double-throw switch, an SE5004L 5.8 GHz power amplifier, and a matched 50 Ω load. The switch operates across 0.1–6 GHz with 62 dB channel isolation and a maximum power handling of 35 dBm. The power amplifier delivers approximately 26 dBm at 5 V. A pulse-width-modulated (PWM) control signal toggles the switch between the amplifier and the load. This operation achieves OOK modulation and reduces the duty cycle of the amplifier. The chain achieves a net gain of approximately 27.5 dB and a saturated output power of 26 dBm. The receiver sensitivity exceeds −65 dBm, and the saturation input power is −45 dBm. An STM32L051 microcontroller generates the modulation waveform and regulates the frame timing. A UHF OOK transceiver module establishes a two-way control link. The radar switches each transponder between operating and standby modes for time-division operation. This link also enables remote monitoring of transponder status and supply voltage. A solar-charged 12 V/20 Ah battery powers the transponder. Pulse modulation maintains the average consumption at several watts.

The main system parameters are listed in Table 1.

The physical origin of the leakage term requires identification prior to the phase error analysis. The system architecture inherently suppresses co-channel carrier leakage. Specifically, the transponder re-transmits the signal at 5.8 GHz. The 5.8 GHz filter in the receive chain rejects the backscattered 2.9 GHz component. Therefore, this component never reaches the mixer. The dominant remaining interference originates solely at the radar end. It comprises two primary sources. The first source is the direct antenna coupling caused by finite transmitter-to-receiver isolation. The second source includes residual leakage and harmonic mixing products from the frequency-multiplied local oscillator path. Internal radar leakage cannot be eliminated by transponder-side doubling. Instead, this leakage couples directly into the local oscillator port of the mixer. Consequently, it interferes coherently with the target echo. This radar-end residual leakage is incorporated into Equations (17)–(19) and restricts the maximum operating distance derived from Equation (19). Both the antenna design and the filters govern this leakage level.

Equation (19) defines the maximum operational distance based on the displacement error threshold. However, the physical limit depends on the two active devices within the chain. The system operates with a transmit power of 33 dBm. The antenna gains are 15 dBi at 2.9 GHz and 20 dBi at 5.8 GHz. The transponder chain integrates a TQPM9037 amplifier, an HMC189A doubler, and an SE5004L power amplifier. This configuration yields a net gain of approximately 27.5 dB and a saturated output power of 26 dBm. The forward loss was 83.9 dB at a distance of 129 m. The transponder input power was −20.9 dBm. This power level exceeded the −45 dBm saturation threshold by 24.1 dB. The return path loss was 89.9 dB. Consequently, the radar received power was −43.3 dBm. This value exceeded the −80 dBm sensitivity by 36.7 dB. The transponder received input powers of −29.5 dBm and −33.8 dBm at distances of 345 m and 570 m, respectively. The corresponding transponder link margins were 15.6 dB and 11.2 dB. The radar received signal powers of −60.4 dBm and −69.1 dBm at these respective distances. The resulting radar link margins were 19.6 dB and 10.9 dB. The transponder received −38.7 dBm at a distance of 1 km. This value provided a 6.3 dB margin above the saturation threshold. The radar received power was −78.9 dBm. This level remained 1.1 dB above the receiver sensitivity limit. The frequency doubler departed from its linear operating region at a distance of 2 km. Therefore, the design distance is jointly constrained by the transponder saturation threshold and the radar receiver sensitivity. Active signal regeneration at the transponder limits the received power decay to the inverse square of the distance rather than the conventional inverse fourth power. This mechanism effectively extends the operational range. This link budget was calculated from measured hardware parameters rather than field measurements. The maximum experimentally verified distance was 570 m. Performance metrics at 1 km represented a theoretical extrapolation.

Equation (17) quantifies the leakage suppression of this architecture. The parameter α denotes the amplitude ratio of the residual leakage to the received echo at the mixer input. The maximum phase error is defined as arcsin(α). The leakage in the conventional continuous-wave radar system falls within the passband of the receive filter. This condition yields an amplitude ratio of 0.8. Consequently, the maximum phase error reaches 53.1 degrees. The resulting displacement error is approximately 3.8 mm at a phase-to-displacement sensitivity of 0.243 rad/mm. The antenna pair in the proposed architecture provides over 50 dB of coupling isolation at 2.9 GHz. The 5.8 GHz filter, together with the more than 30 dB fundamental and harmonic suppression of the HMC189A doubler, provides over 80 dB of combined suppression. These stages reduce the amplitude ratio to approximately 0.005. Accordingly, the maximum phase error decreases to 0.3 degrees, and the displacement error drops to approximately 0.02 mm. This design suppresses the leakage-induced displacement error by more than two orders of magnitude. The final error satisfies the 0.8 mm design specification with substantial margin.

#### 2.5.2. Field Experimental Setup

Field electromagnetic wave propagation is subject to atmospheric disturbance, vegetation occlusion, and structural reflections. A natural slope site was selected for field testing. This environment validated the system’s adaptability under complex field conditions. The experiments were conducted under three typical scenarios: an unobstructed line-of-sight (LOS) at 129 m, slight occlusion at 345 m, and dense shrub occlusion at 570 m. Additionally, a synchronous dual-target experiment at 129 m was conducted to evaluate the multi-target displacement monitoring capability of the system.

**Figure 4:**
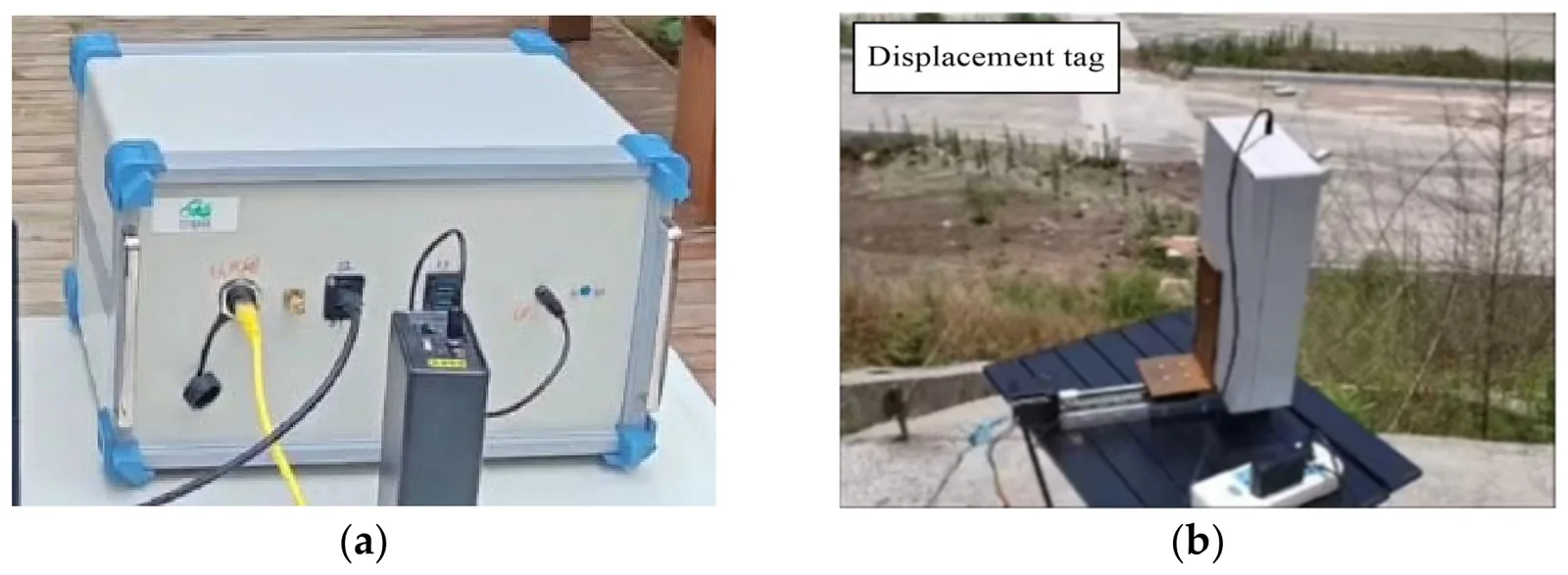
Hardware configuration of the monitoring system: (**a**) radar monitoring platform; (**b**) displacement monitoring target. Both units are deployed at the field test site illustrated in Figure 5.

Direct measurement of continuous deformation in actual landslides or large structures presents practical difficulties. Consequently, the transponder was mounted on an electric linear stage driven by a remotely commanded stepper motor. This remote configuration eliminated the need for operator proximity during measurements. It also prevented phase interference caused by human activity. Figure 4 illustrates the hardware deployment of both terminals. Figure 4a shows the radar monitoring platform. This platform integrates RF transmission, signal reception, data acquisition, and host processing. Figure 4b displays the target terminal. This unit comprises the transponder, a dual-band planar antenna, and the linear stage. The linear stage provides traceable reference displacements. However, it cannot replicate the distributed deformation patterns of actual slopes. Long-term field monitoring of real structures remains an objective for future work.

**Figure 5:**
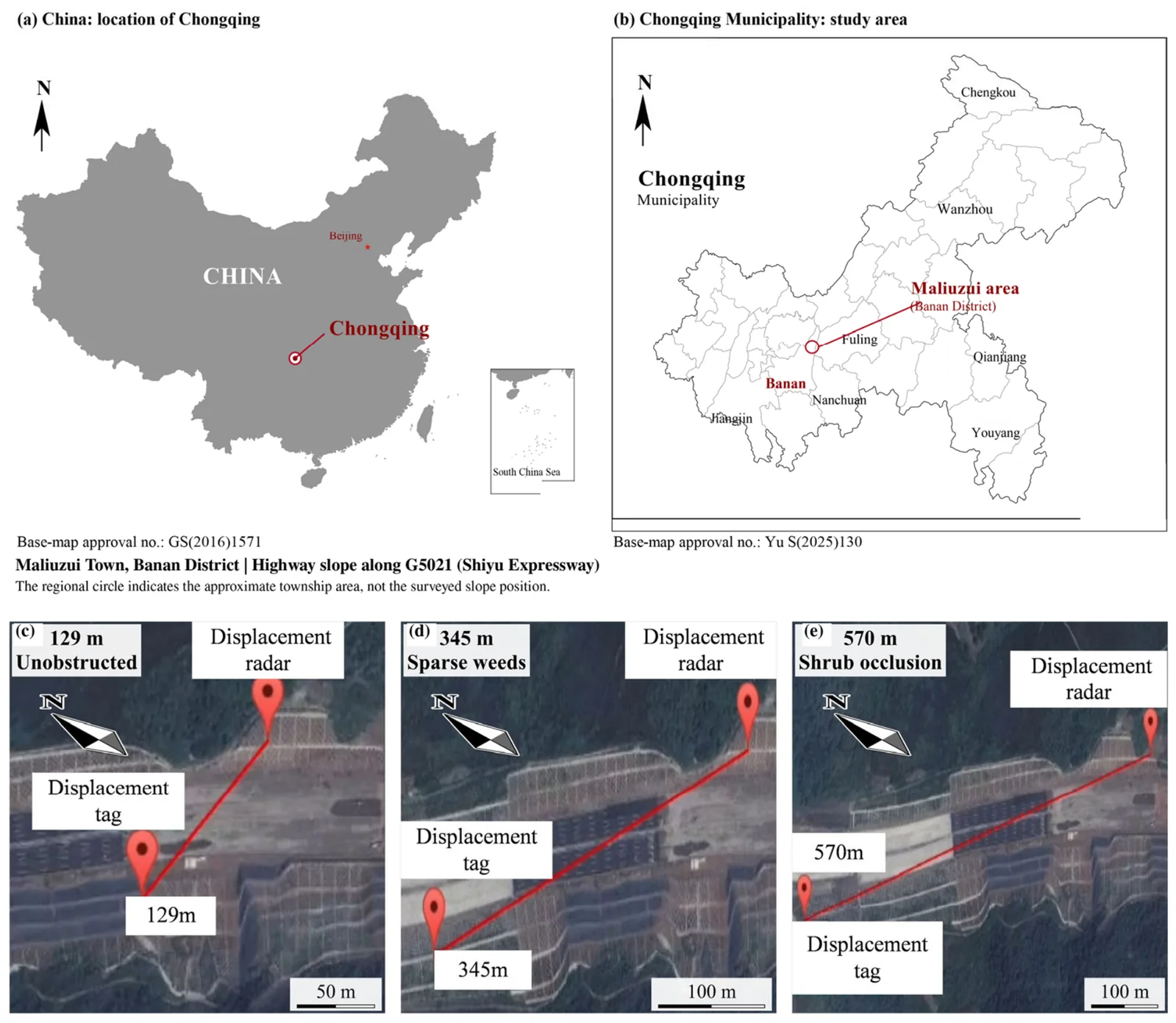
Geographic location and field deployment of the slope experiment in Maliuzui Town, Banan District, Chongqing, China, along the G5021 (Shiyu Expressway): (**a**) location of Chongqing in China; (**b**) regional location of the study area in Chongqing Municipality; (**c**) 129 m unobstructed test; (**d**) 345 m slight occlusion test with sparse weeds; and (**e**) 570 m shrub occlusion test. The circle in (**b**) indicates the approximate township area, not a surveyed coordinate of the test slope. The base maps in (**a**,**b**) were obtained from the Ministry of Natural Resources and Chongqing Standard Map Services, respectively. Base-map approval numbers are GS(2016)1571 and Yu S(2025)130, respectively. English labels and location markers have been added; geographic outlines are retained. In (**c**–**e**), the red lines connect the radar and transponder (displacement tag), and the scale bars are derived from the measured line-of-sight distances. At mid-path, the first Fresnel zone radii are approximately 1.8/3.0/3.8 m at 2.9 GHz and 1.3/2.1/2.7 m at 5.8 GHz for the three distances, respectively.

Figure 5c–e illustrate the deployment configurations for the three experimental scenarios. These configurations correspond to short-range unobstructed, medium-range slightly occluded, and long-range vegetation-occluded environments, respectively. The first Fresnel zone remained unobstructed in the 129 m scenario. Consequently, the radar echo experienced no diffraction loss. Sparse vegetation obstructed the lower boundary of the Fresnel zone in the 345 m scenario. This obstruction introduced minor signal attenuation. A shrub layer intercepted the propagation path in the 570 m scenario. Dynamic canopy motion induced continuous fluctuations in both the amplitude and phase of the echo. The radar station was mounted on a stable platform to eliminate phase errors from self-motion. The transponder was positioned on a displacement stage at the target monitoring point. All three values represented direct LOS distances measured at a highway-slope test site along the G5021 Shiyu Expressway in Maliuzui Town, Banan District, Chongqing, China.

The single-step stage displacement was set to 2.5 mm. The system performance was evaluated through displacement curve consistency, step identification capability, measurement error, maximum absolute error, and root mean square error (RMSE). The corresponding experimental results are presented in the Results section.

## 3. Results

### 3.1. System Feasibility Simulation

**Figure 6:**
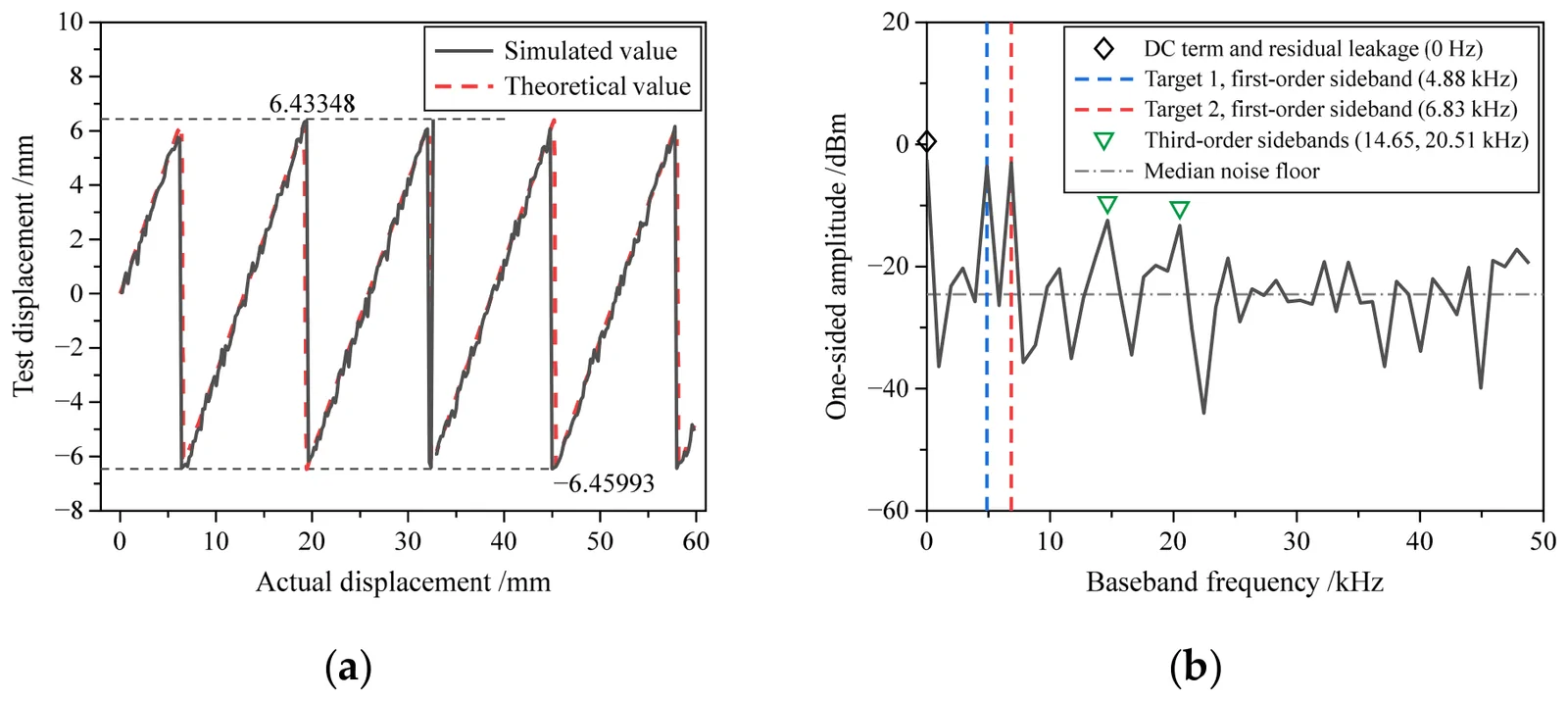
System feasibility simulation results: (**a**) simulated displacement; (**b**) amplitude spectrum. The horizontal axis in Figure 6b represents the baseband frequency in kHz. A 1024-point FFT at a sampling rate of 1 MHz yields a spectral resolution of 976.6 Hz. The vertical axis denotes the one-sided amplitude in dBm. The full-scale input of the ADC serves as the 0-dBm reference level. The two OOK modulation frequencies are 4.88 kHz and 6.83 kHz. The legend identifies the DC term, the first-order sidebands of both targets, the third-order sidebands, and the median noise floor.

A dual-target separation simulation was conducted to evaluate the frequency-division identification capability of the system. The simulation employed a 1024-point FFT at a sampling frequency of 1 MHz. This configuration yielded an adjacent spectral line spacing of 976.6 Hz. The OOK modulation frequencies were set to 4.88 kHz and 6.83 kHz with a separation of two spectral lines. The simulated spectrum in Figure 6 contains four distinct component groups. The zero-frequency component represents the DC term generated by local oscillator self-mixing and residual radar leakage. This component carries no displacement information. The signals at 4.88 kHz and 6.83 kHz correspond to the first-order sidebands of the two transponders. This two-line separation between these primary signals prevents mutual leakage. The remaining spectral components consist of third-order sidebands and the receiver noise floor. The sidebands at 14.65 kHz and 20.51 kHz are approximately 9–10 dB below the primary signal peaks. The median receiver noise floor is approximately 21 dB below these peaks. The phase at 4.88 kHz was used for the displacement solution (Figure 6a). This result demonstrates strong agreement with the theoretical curve. This consistency confirms the capability for simultaneous and independent multi-target measurements. The 1024-point observation window spans 1.024 ms. This duration is significantly shorter than the decorrelation time of a vegetated propagation path. Consequently, canopy motion manifests as a slow phase drift rather than an elevated noise floor within a single observation. The simulation parameters were selected to match the realized hardware one-to-one. The sampling frequency of 1 MHz equals the maximum conversion rate of the AD9226 12-bit ADC described in Section 2.5.1, and the 1024-point FFT is the transform length implemented in the FPGA, which establishes the spectral resolution of 976.6 Hz. The two OOK frequencies are generated by the STM32L051 controller of each transponder, so they are integer multiples of this resolution: 4.88 kHz corresponds to 5 bins and 6.83 kHz to 7 bins. The third-order sidebands at 14.65 kHz and 20.51 kHz correspond to 15 and 21 bins, respectively.

### 3.2. Field Experiment Results

Field experiments were conducted under three representative conditions: 129 m unobstructed LOS, 345 m slight occlusion, and 570 m shrub occlusion. A dual-target synchronous displacement monitoring experiment was also performed at 129 m. The single-step displacement of the linear stage was fixed at 2.5 mm for all experiments.

#### 3.2.1. Displacement Monitoring Under Different Scenarios

**Figure 7:**
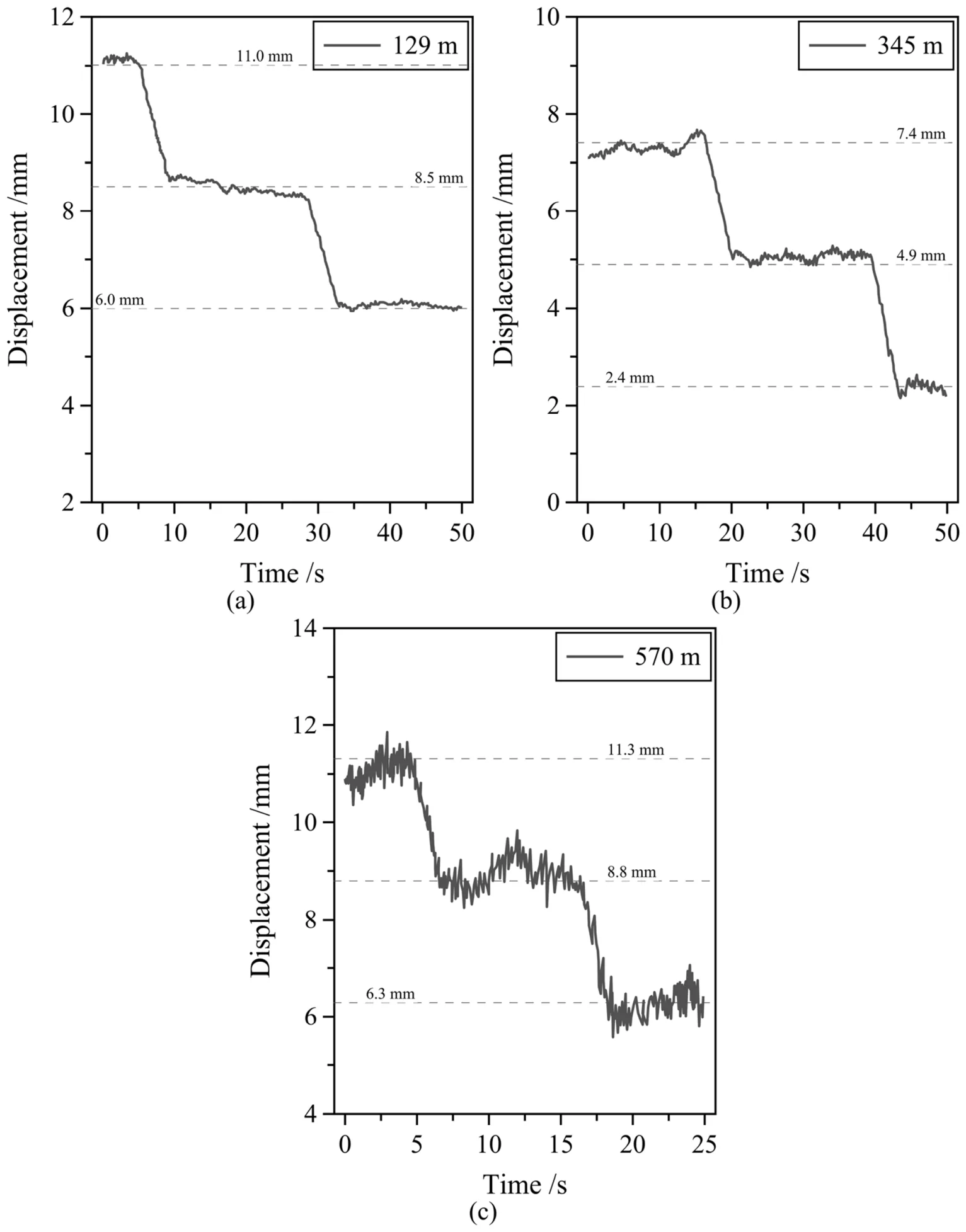
Displacement curves from the slope experiment: (**a**) 129 m unobstructed test; (**b**) 345 m slight occlusion test; (**c**) 570 m shrub occlusion test. All vertical axes share a uniform 10 mm range. This consistent scale enables direct comparison of the fluctuations.

Figure 7 illustrates the displacement monitoring results across different experimental scenarios. The system generated distinct displacement curves for both the 129 m unobstructed condition and the 345 m slight occlusion condition. The observed displacement step points agree closely with the theoretical values. The 2.5 mm micro-displacement variations were reliably identified. These results demonstrate high measurement stability and robust phase detection performance under short- and medium-range conditions.

Shrub occlusion at a 570 m range introduces vegetation scattering, multipath effects, and environmental noise. These factors caused echo waveform distortions and localized displacement fluctuations. Nevertheless, the system reliably identified the 2.5 mm displacement trend. This outcome confirms the robust long-range micro-deformation monitoring capability of the proposed frequency-multiplication transponder in complex environments.

#### 3.2.2. Quantitative Measurement Error Analysis

**Figure 8:**
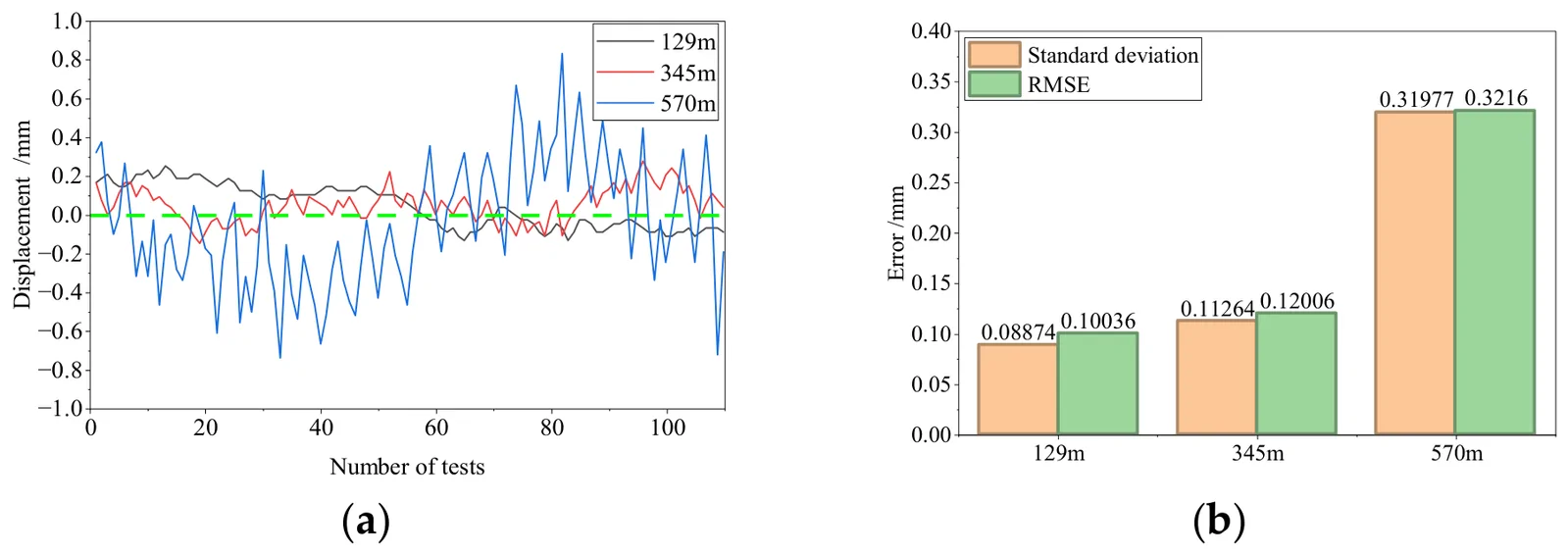
Displacement errors in the slope experiment: (**a**) raw error; (**b**) standard deviation and RMSE.

Figure 8 presents the displacement error statistics under different scenarios. The error remained stable at approximately 0.1 mm under the 129 m unobstructed condition. It increased slightly under the 345 m slight occlusion condition. The error exhibited higher fluctuation under the 570 m shrub occlusion condition. This increase occurred because the echo signal approached the receiver sensitivity threshold. Furthermore, vegetation introduced amplitude and phase scintillation. Nevertheless, the maximum absolute error remained below 0.8 mm, and the RMSE was 0.32 mm. The residual error represents the root sum of squares of three components. These components comprise the reference stage positioning error, the environmental phase noise, and the front-end noise term. Environmental noise stems from vibration, multipath effects, and vegetation dynamics. The front-end term consists of thermal noise and residual leakage. The stage positioning error and environmental phase noise remained independent of the distance. Specifically, the stage error introduces a fixed offset of approximately 0.06 mm. Environmental phase noise contributes an offset of approximately 0.13 mm. In contrast, the front-end term increases proportionally with measurement distance. Therefore, environmental phase noise dominated the error at 129 m rather than the receiver noise. The front-end noise term at 570 m increased to approximately 0.29 mm. This term accounts for the majority of the measured RMSE. This increase aligns with the SNR in the OOK bin, which decreased from approximately 73 dB at 129 m to 56 dB at 570 m. A 17 dB reduction in the SNR modulation line alone would raise the phase noise by a factor of about seven. This corresponded to an increase from 0.04 mm at 129 m to about 0.29 mm at 570 m. In contrast, the measured error increased by only a factor of approximately two. Consequently, environmental phase fluctuations along the occluded path caused the additional error at 570 m rather than the reduced SNR. The radar front end accounted for roughly 70% of the total error power at 570 m. This contribution decreased to approximately 20% at 129 m. Phase extraction utilized this single spectral bin. The modulation energy was concentrated within the first-order sidebands. This concentration elevated the effective SNR substantially above the full-bandwidth value. An extrapolation of the front-end term to the design distance of 1 km yielded a projected RMSE of about 0.52 mm. This projected value remained below the 0.8 mm specification. This reported value represents an extrapolation estimate rather than a direct measurement. Each term of this budget was derived from the recorded data rather than assumed. The stage term originates from the positioning repeatability of the linear stage that supplies the reference displacements (Section 2.5.2). The environmental term was obtained from a zero-displacement interval recorded with the transponder at rest, in which the standard deviation of the extracted phase series is converted to displacement through Equation (16). The front-end term was isolated as the residual error power after the two distance-independent contributions were removed, and it was cross-checked against the phase noise predicted from the measured SNR in the modulation bin through Equation (9). The root sum of squares of the three terms is 0.32 mm, which reproduces the measured RMSE at 570 m and therefore confirms the consistency of the decomposition.

Measurement error increased with monitoring distance and environmental complexity. The system consistently maintained sub-millimeter accuracy. These results confirm the engineering feasibility of the proposed radar in complex field environments.

### 3.3. Dual-Target Synchronous Monitoring Results

**Figure 9:**
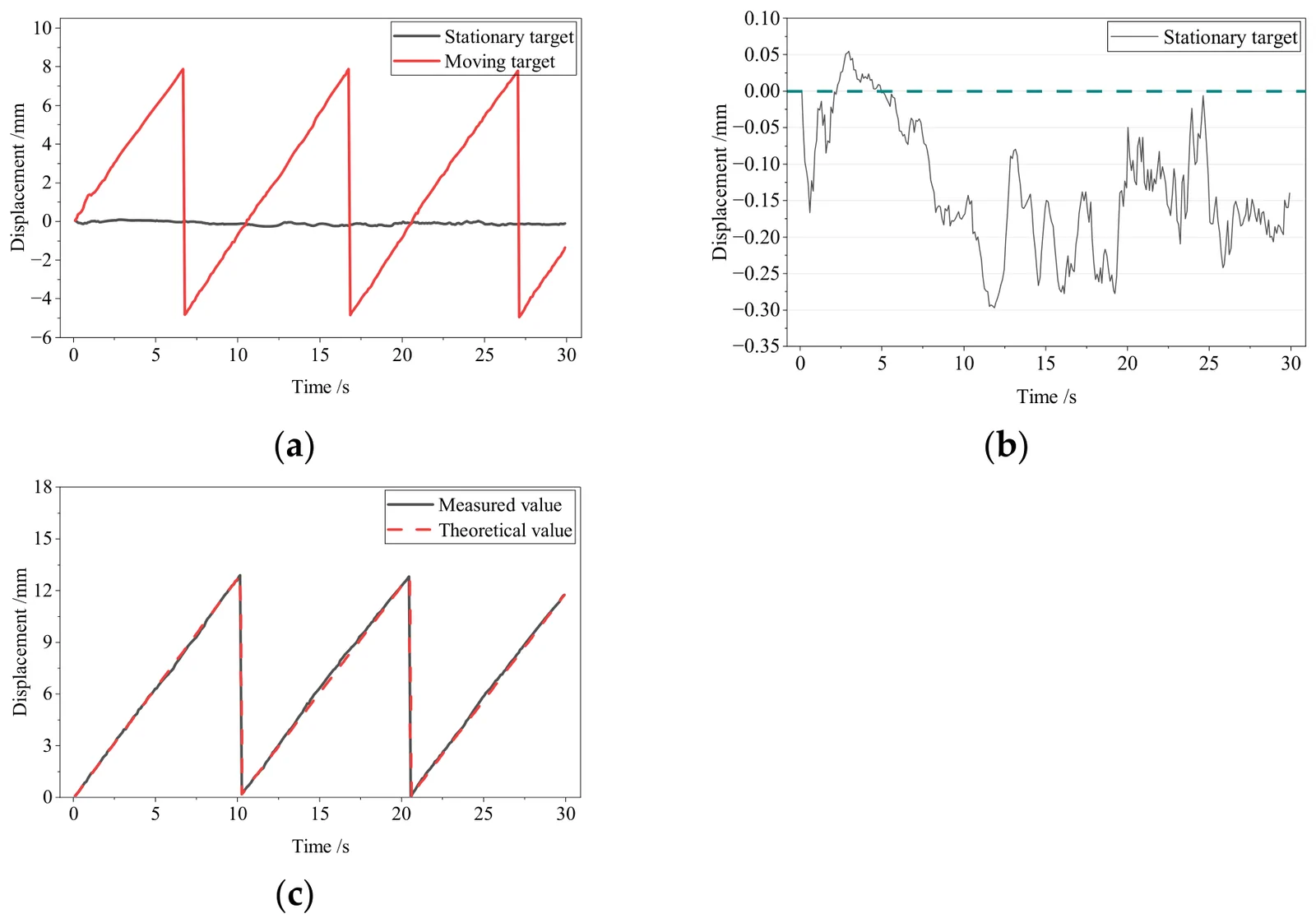
Dual-target displacement monitoring results: (**a**) measured dual-target displacement; (**b**) Stationary target; (**c**) Moving target.

Figure 9 shows the dual-target synchronous displacement monitoring results at 129 m. The two monitoring targets were encoded with different OOK modulation frequencies. FFT processing decoupled the superimposed target echoes in the frequency domain. The displacement of each target was extracted independently.

The stationary target exhibited a stable displacement profile with minor random fluctuations. The tracked trajectory of the moving target aligned closely with the motion of the linear stage. The dual-target synchronous monitoring yielded a maximum measurement error below 0.4 mm. The RMSE was 0.14 mm. The two targets in this experiment returned comparable echo powers, so the near-far problem did not occur here. In OOK frequency division, a power disparity between targets is carried into the amplitudes of their modulation sidebands within the same received spectrum. When one transponder is much closer, its first-order sidebands and the spectral leakage of the finite observation window raise the level in the neighbouring bins and can mask the weaker target. Its third-order sidebands, observed here at 14.65 kHz and 20.51 kHz, may also approach the first-order line of a weak target. Three measures are adopted against this effect. The modulation frequencies are placed on integer multiples of the FFT bin and separated by at least two bins, so that the first-order lines of different targets never share a bin. A PE43703 digital step attenuator in the receive chain provides 0 to 30 dB of level adjustment, which keeps both returns within the input range of the ADC. The duty cycle of the transponder power amplifier is limited by the PWM drive, which suppresses the high-order sidebands. For power disparities beyond what these measures cover, time-division scheduling through the UHF control link provides a fallback.

### 3.4. Comparison with Representative Interferometric Radar Systems

The proposed system achieved high-accuracy displacement monitoring at 570 m. It simultaneously monitored multiple targets. The instrument operates as a sparse point-type device. Each target location requires an active transponder. The measurements capture only radial displacements along the line of sight. Therefore, the system is not a full-field imaging platform. It does not generate continuous regional deformation maps. In contrast, frequency-modulated continuous-wave ground-based interferometric synthetic aperture radar (GB-InSAR) and multiple-input multiple-output synthetic aperture radar (MIMO-SAR) provide full-field imaging capabilities. These imaging radars yield high spatial point density across a wide field of view. However, these systems entail substantial hardware complexity and high implementation costs. Consequently, the proposed scheme serves as a targeted complement to imaging radars rather than a direct substitute. Both approaches achieved sub-millimeter displacement resolution. The interferometric phase determined this resolution instead of the waveform. Imaging radars provide a range resolution of 0.5 m to 1 m across the entire field of view. In contrast, the proposed system lacked range resolution and monitored only instrumented points. The proposed system achieved update rates of up to 10 Hz for each point. GB-InSAR requires several minutes per acquisition. Furthermore, the proposed system unambiguously separates multiple co-range targets via frequency division. Conventional continuous-wave interferometric radar cannot achieve such separation. The single-channel architecture and largely passive transponders maintain low system complexity and low per-point costs. However, each monitoring point requires an independent power supply. Strong transponder echoes substantially suppress the atmospheric phase disturbances that degrade continuous-wave systems. Table 2 summarizes the performance comparison among representative systems.

Conventional continuous-wave (CW) interferometric radar [26] features a simple architecture for short-range micro-displacement monitoring. However, its multi-target identification capability remains limited. The GB-InSAR [27] using frequency-modulated continuous wave (FMCW) provides high range resolution. Nevertheless, this system exhibits high structural complexity. The MIMO-SAR [28] and millimeter-wave (mmWave) interferometric radar [29] offer fine spatial resolution and high-frequency sensitivity. However, these systems face constraints on multi-target synchronization and practical engineering applications. In contrast, the proposed frequency-multiplication transponder interferometric radar overcomes these limitations. It achieves long-range detection, sub-millimeter precision, and simultaneous multi-target monitoring. These capabilities rely on transmit-receive frequency isolation, OOK frequency-division multi-target demodulation, and echo enhancement. Furthermore, the overall system architecture maintains low complexity. Consequently, this radar presents an effective solution for engineering micro-deformation monitoring.

## 4. Conclusions

This study proposed a deformation monitoring radar utilizing a frequency-multiplication transponder. The system targets long-range micro-deformation monitoring of geological hazards and large infrastructure. Frequency multiplication between the transmitted and received signals eliminates transmitter leakage. This mechanism substantially improves phase measurements for weak displacement signals. Active frequency-multiplication amplification enhances the long-distance echo power. In addition, OOK frequency-division modulation enables synchronous identification and demodulation across multiple targets. An interferometric measurement model was established to derive the relationship between target displacement and echo phase variation. Field experiments confirmed reliable detection of 2.5 mm micro-displacements. The measurement error reached approximately 0.1 mm under the 129 m unobstructed condition. The maximum absolute error remained below 0.8 mm in complex environments up to 570 m. Synchronous displacement demodulation yielded an RMSE of 0.14 mm in the 129 m dual-target test. These results indicated that the proposed radar maintained sub-millimeter accuracy over long distances and provided robust multi-target monitoring capabilities. The 1 km design range quoted in this work is a theoretical limit derived from the measured link budget; the maximum distance verified experimentally is 570 m.

The proposed system enables early micro-deformation monitoring for natural slopes, engineered slopes, and landslides. Its application requires the consideration of a sparse point-type measurement layout. Transponders are installed along the landslide cross-section. The radar station is positioned in a stable reference area. This configuration measures radial displacement along the primary sliding direction. Effective early warning requires two technical prerequisites. First, the sensor specifications must match the target deformation rates. The system provides sub-millimeter accuracy and a sampling frequency of up to 10 Hz. These parameters allow the detection of progressive pre-failure deformation occurring over hours to months. However, the system cannot detect sudden collapse occurring within a single sampling interval. The methodology therefore targets progressive deformation monitoring rather than instantaneous failure prediction. Second, site-specific displacement rate thresholds and alarm criteria are mandatory. These parameters depend on local geological conditions and historical displacement trends. The measurement reflects surface displacement rather than slope stability directly.

The current system exhibits two primary limitations. First, the maximum effective monitoring range requires comprehensive experimental verification. Second, measurement stability under strong electromagnetic interference, dense vegetation, and complex multipath conditions demands enhancement. Future research will address these challenges through targeted investigations. System transmit power and antenna gain will be increased to evaluate kilometer-level monitoring performance. Anti-interference algorithms and digital filtering mechanisms will be optimized for harsh operating environments. Long-term field trials will be deployed on representative structures, including bridges, dams, and steep slopes. These trials will establish standardized engineering application protocols and technical specifications.

## Figures and Tables

**Table 1:** Main parameters of the radar system.

Parameter	Value	Parameter	Value
Transmit frequency	2.9 GHz	Measurement accuracy	±0.8 mm; RMSE ≤ 0.32 mm
Receive frequency	5.8 GHz	Sampling rate	≤1 MSps
Verified monitoring distance	570 m	Monitoring frequency	≤10 Hz
Modulation scheme	OOK	Number of targets	≥2
Bandwidth	1 MHz	Design range (link budget)	1 km

**Table 2:** Comparison of representative interferometric radar systems. Note: N/R denotes not reported in the cited literature.

System Category	Radar Regime	Core Interferometric Mechanism	System Features	System Limitations	Reported Performance
Continuous-wave phase-interferometric radar [26]	CW, MIMO	Single-frequency phase-difference interferometry	Simple; real-time micro-displacement measurement	No range resolution; difficult multi-target separation	Band: 77 GHz
FMCW-GB-InSAR [27]	FMCW, GB-SAR	FMCW range compression and interferometric phase analysis	Combines range resolution with micro-deformation measurement; suitable for slopes and structures	Complex SAR imaging chain; high real-time processing load	Band: N/R
MIMO-SAR interferometric radar [28]	MIMO-SAR	Synthetic (virtual) aperture interferometry	No mechanical scanning; high refresh rate; wide field of view	Complex multi-channel synchronization and calibration; strict coherence	Band: 76–81 GHz
Millimeter-wave interferometric radar [29]	mmWave radar	High-frequency phase interferometry	Short wavelength; high displacement sensitivity; miniaturization potential	Sensitive to atmospheric phase disturbance; limited long-term stability	Band: 79 GHz
Proposed frequency-multiplication transponder interferometric radar	Frequency-offset multiplication transponder, CW	Frequency-offset transponder phase interferometry	Suppresses leakage; enhances echo; OOK supports multi-target monitoring; simple structure	Active transponder requires power; spectral leakage between transponder signals	Band: 2.9/5.8 GHz

## Data Availability

The data presented in this study are available on request from the corresponding author.

## Abbreviations

The following abbreviations are used in this manuscript:

A/D	Analog-to-digital
CW	Continuous wave
FFT	Fast Fourier transform
FPGA	Field-programmable gate array
FMCW	Frequency-modulated continuous wave
GB-InSAR	Ground-based interferometric synthetic aperture radar
GNSS	Global navigation satellite system
IF	Intermediate frequency
I/Q	In-phase/quadrature
LNA	Low noise amplifier
LOS	Line-of-sight
MCU	Microcontroller unit
MIMO-SAR	Multiple-input multiple-output synthetic aperture radar
mmWave	Millimeter wave
OOK	On–off keying
RF	Radio frequency
RMSE	Root mean square error
SNR	Signal-to-noise ratio
